# Carbon nanotube/Co_3_O_4_ nanocomposites selectively coated by polyaniline for high performance air electrodes

**DOI:** 10.1038/s41598-017-09219-9

**Published:** 2017-08-17

**Authors:** Jin Young Kim, Yong Joon Park

**Affiliations:** Department of Advanced Materials Engineering, Kyonggi University, 154-42, Gwanggyosan-Ro, Yeongtong-Gu, Suwon-Si, Gyeonggi-Do Republic of Korea

## Abstract

We herein report the preparation of carbon nanotube (CNT)/Co_3_O_4_ nanocomposites selectively coated with polyaniline (PANI) via an electropolymerization method, for use as an effective electrode material for Li-air (Li-O_2_) batteries. The Co_3_O_4_ catalyst attached to the CNTs facilitated the dissociation of reaction products and reduced the overpotential of the cells. As the carbon surface activates the side reactions, the PANI coating on the carbon surface of the electrode suppressed the side reaction at the electrode/Li_2_O_2_ and electrode/electrolyte interfaces, thus enhancing the cycle performance of the electrode. In addition, the catalytic activity of Co_3_O_4_ on the CNT/Co_3_O_4_ nanocomposites remained unaffected, as the Co_3_O_4_ surface was not covered with a PANI layer due to the nature of the electropolymerization method. Overall, the synergic effect of the PANI layer and the Co_3_O_4_ catalyst leads to a superior cyclic performance and a low overpotential for the electrode based on selectively PANI-coated CNT/Co_3_O_4_ nanocomposites.

## Introduction

In recent decades, the worldwide increase in environmental concerns has triggered the development and commercialization of electrified transportation systems, such as electric vehicles (EV), which commonly employ lithium-ion batteries. However, the short driving range of EVs, attributed to the insufficient energy densities of current batteries, has limited effective competition with gasoline-powered cars. As such, the exploration of novel battery chemistries to achieve high energy storage capacities is a growing priority for research and development. For example, non-aqueous Li-air (practically Li-O_2_) batteries represent one of the most appealing candidates for replacing lithium-ion batteries, because their achievable energy densities are expected to be several times higher than those of the most advanced lithium-ion batteries^1–7^. However, these batteries suffer from several issues, including limited cycle lives, low energy efficiencies, and high cell polarizations (over-potential)^1–7^.

Li-air (Li-O_2_) batteries operate through a complex reaction mechanism, which involves the reduction of molecular oxygen in an organic electrolyte, and subsequent formation of reaction products such as Li_2_O_2_ (lithium peroxide) at the electrode/electrolyte interface upon discharge^8–15^. Thus, to obtain stable cycle performances and high energy efficiencies, these reaction products should be perfectly dissociated to yield molecular oxygen and lithium ions during charging with a low overpotential^8–21^. However, such dissociation requires a high energy, due to the non-conducting nature of Li_2_O_2_, which results in low energy efficiencies and high overpotentials for the Li-air (Li-O_2_) cells^22–27^. Moreover, unwanted reaction products, such as Li_2_CO_3_ and organic materials (e.g., CH_3_CO_2_Li and HCO_2_Li), which are barely dissociated even at high overpotentials, are gradually accumulated on the surface of the air electrode during cycling. In addition, the highly reactive superoxide decomposes the electrolyte, while the carbon materials in the air electrode activate the side-reaction at the Li_2_O_2_/carbon interphase and oxidize the electrolyte, thus leading to the formation of unwanted side-products^28–35^. This accumulation of unwanted by-products results in clogging of the electrode, and ultimately limits the cycle performance of the Li-air (Li-O_2_) cells^22–35^.

It has been reported that the application of catalysts based on noble metals^36–40^ and metal oxides^41–47^ to the air electrode plays a vital role in the dissociation of reaction products, with several noble metals successfully reducing the over-potential of Li-air (Li-O_2_) cells. However, these species inevitably promote other side reactions, including decomposition of the electrolyte solution and of carbon, due to their unselective catalytic activity. In contrast, metal oxides (e.g., MnO_2_, Co_3_O_4_, and LaMnO_3_) have been employed as catalysts in air electrodes because of their low costs and reliable catalytic activities^41–47^. Moreover, the side reactions attributed to metal oxide catalysts are relatively small^48, 49^. In many cases, metal oxides are combined with carbon matrices to compensate for their low electronic conductivities and to homogenously disperse the nano-sized catalyst particles on the surface of the air electrode^45–47^. Despite the success of such catalysts, limited cycle performances remain an issue for Li-air (Li-O_2_) cells containing metal oxide catalysts, as the side reactions involving superoxides and the electrode carbon materials cannot be suppressed by the catalysts.

**Figure 1:**
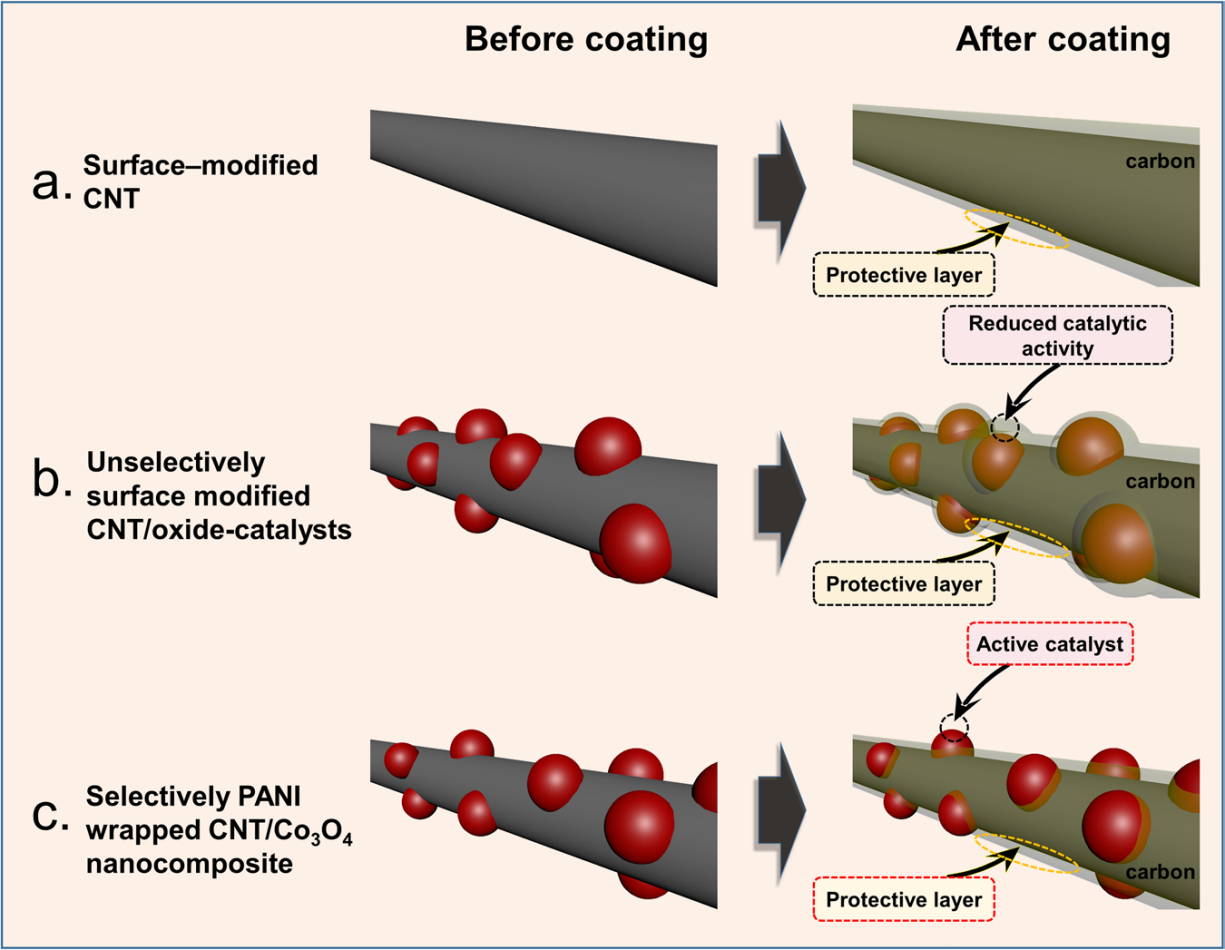
Schematic diagram showing (**a**) the surface-modified CNT, (**b**) the unselectively surface-modified CNT/oxide-catalysts, and (**c**) the selectively PANI-coated CNT/Co_3_O_4_ nanocomposite.

To address this issue, we previously reported modification of the carbon surface of the air electrode using a stable polymer, as shown in Fig. 1a ^50–53^. More specifically, Li-air (Li-O_2_) cells containing a carbon surface coated with polydopamine, polyimide, or poly(3,4-ethylenedioxythiophene) polystyrene sulfonate (PEDOT:PSS) exhibited enhanced cycle performances compared to the cells employing pristine carbon materials. This difference was therefore attributed to the thin surface coating layer limiting direct contact between carbon and the electrolyte and/or the reaction products, which in turn inhibited carbon-activated side reactions. However, this polymer coating was unselectively formed on the surface of the air electrode. We therefore expect that the application of a polymer coating to the carbon/oxide-catalyst composites would result in coating of both the carbon surface and the catalyst surface with the polymer layer (Fig. 1b). These methods therefore inevitably lead to a decrease in catalytic activity for such composites, despite the polymer coating suppressing the carbon-promoted side reactions.

Thus, we herein report the selective coating of the surfaces of carbon nanotube (CNT)/Co_3_O_4_ nanocomposites with a polyaniline (PANI) layer using an electropolymerization method, as illustrated in Fig. 1c. We expect that although the CNT surface can be coated with PANI prepared from aniline monomers via an electropolymerization procedure, the Co_3_O_4_ catalyst surface will remain uncoated due to the non-conducting nature of this surface. Furthermore, PANI is of particular interest for this application, as it is a stable polymer material that offers high electronic conductivity^54–56^, and so it should facilitate the redox reaction between lithium ions and oxygen species, whilst also protecting the carbon surface against the electrolyte and/or the reaction products. Moreover, the Li-air (Li-O_2_) cells employing these selectively-coated CNT/Co_3_O_4_ nanocomposites are expected to exhibit enhanced cyclic performances due to suppression of the carbon-based side-reactions without any reduction in the Co_3_O_4_ catalytic activity.

## Results and Discussion

**Figure 2:**
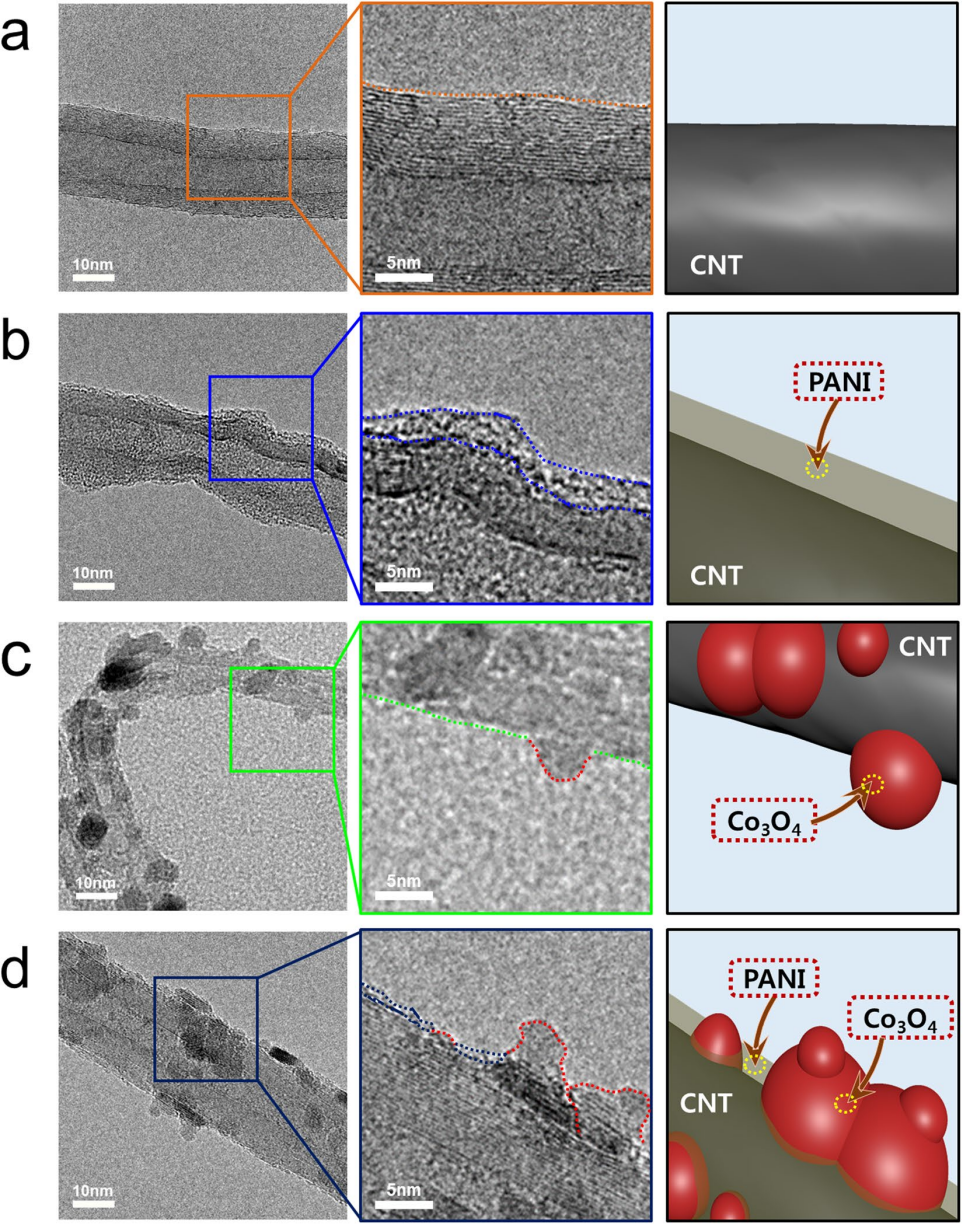
TEM images of (**a**) the pristine CNTs, (**b**) the PANI-coated CNT, (**c**) the CNT/Co_3_O_4_ nanocomposite, and (**d**) the selectively PANI-coated CNT/Co_3_O_4_ composite.

To examine the morphologies of the pristine CNTs, the PANI-coated CNTs, the CNT/Co_3_O_4_ composite, and the selectively PANI-coated CNT/Co_3_O_4_ composite, TEM was carried out. As shown in Fig. 2a, the pristine CNT exhibited a structure typical of a multi-walled CNT with smooth side walls. No heterogeneous particles or layers were observed on the surface.

In contrast, the surface of the PANI-coated CNT was clearly covered by a thin layer of 2–4 nm thickness, which was expected to be the PANI coating (Fig. 2b). As shown in Fig. 2c, the surface of the CNT/Co_3_O_4_ composite was homogeneously modified with Co_3_O_4_ nanoparticles measuring several nanometers in diameter. These particles are expected to act as efficient catalysts because of their large catalytic active layer, which could be attributed to their high surface area and good electron transportation properties through the CNT. The selectively PANI-coated CNT/Co_3_O_4_ composite contained both a PANI layer on the surface of the CNT wall and homogenously dispersed Co_3_O_4_ nanoparticles on the CNT (Fig. 2d). Interestingly, the Co_3_O_4_ particle surface was not coated with PANI, as the electropolymerization method employed herein allows deposition only on the surface of electronic conductors. As such, the TEM images shown in Fig. 2d confirm the selective coating of the CNT/Co_3_O_4_ composite with a PANI layer.

Formation of the PANI layer was also confirmed by FTIR spectroscopy, as shown in Figure S1. More specifically, the PANI-coated CNT exhibited an intense signal at approx. 1130 cm^−1^, which could be attributed to the C-H bonds of PANI. Several broad peaks were also present at 1301, 1506, and 1592 cm^−1^, which originated from the C-N, C=C (benzenoid stretching), and C=C (quinonoid stretching) bonds of PANI^57, 58^. These results therefore confirm that the PANI layer was successfully formed on the CNT surface.

To determine the effects of the PANI layer and the Co_3_O_4_ catalyst, the electrochemical properties of Li-air (Li-O_2_) cells based on pristine CNTs, PANI-coated CNTs, CNT/Co_3_O_4_ composites, and selectively PANI-coated CNT/Co_3_O_4_ composites were observed and compared. For convenience, the electrode containing the pristine CNTs will hereafter be referred to as the ‘pristine electrode’, the electrode employing the PANI-coated CNTs will be referred to as the ‘PANI electrode’, the electrode based on the CNT/Co_3_O_4_ composite will be known as the ‘comp electrode’, and the electrode containing the selectively PANI-coated CNT/Co_3_O_4_ composites will be referred to as the ‘PANI-comp electrode’. In this context, Figure S2 shows the initial full discharge-charge profile of the cells employing the four different electrodes at a current density of 500 mA·g_electrode_^−1^. The full discharge capacity of the pristine electrode was approx. 12000 mAh·g_electrode_^−1^ (Figure S2a), while that of the PANI electrode was somewhat lower (i.e., approx. 8000 mAh·g_electrode_^−1^, see Figure S2b). This is likely due to the PANI coating decreasing the electrode surface area in comparison to that of the carbon-based electrode. The comp electrode also exhibited a lower discharge capacity (approx. 7800 mAh·g_electrode_^−1^) than the pristine electrode because of the higher mass of the oxide catalyst (Co_3_O_4_) compared to the CNTs (Figure S2c). In addition, the discharge capacity of the PANI-comp electrode was rather low (i.e., approx. 4200 mAh·g_electrode_^−1^, Figure S2d), likely due to the synchronous effect of the Co_3_O_4_ nanoparticles and the PANI layer. However, the overpotential appeared lower than that of the pristine electrode.

**Figure 3:**
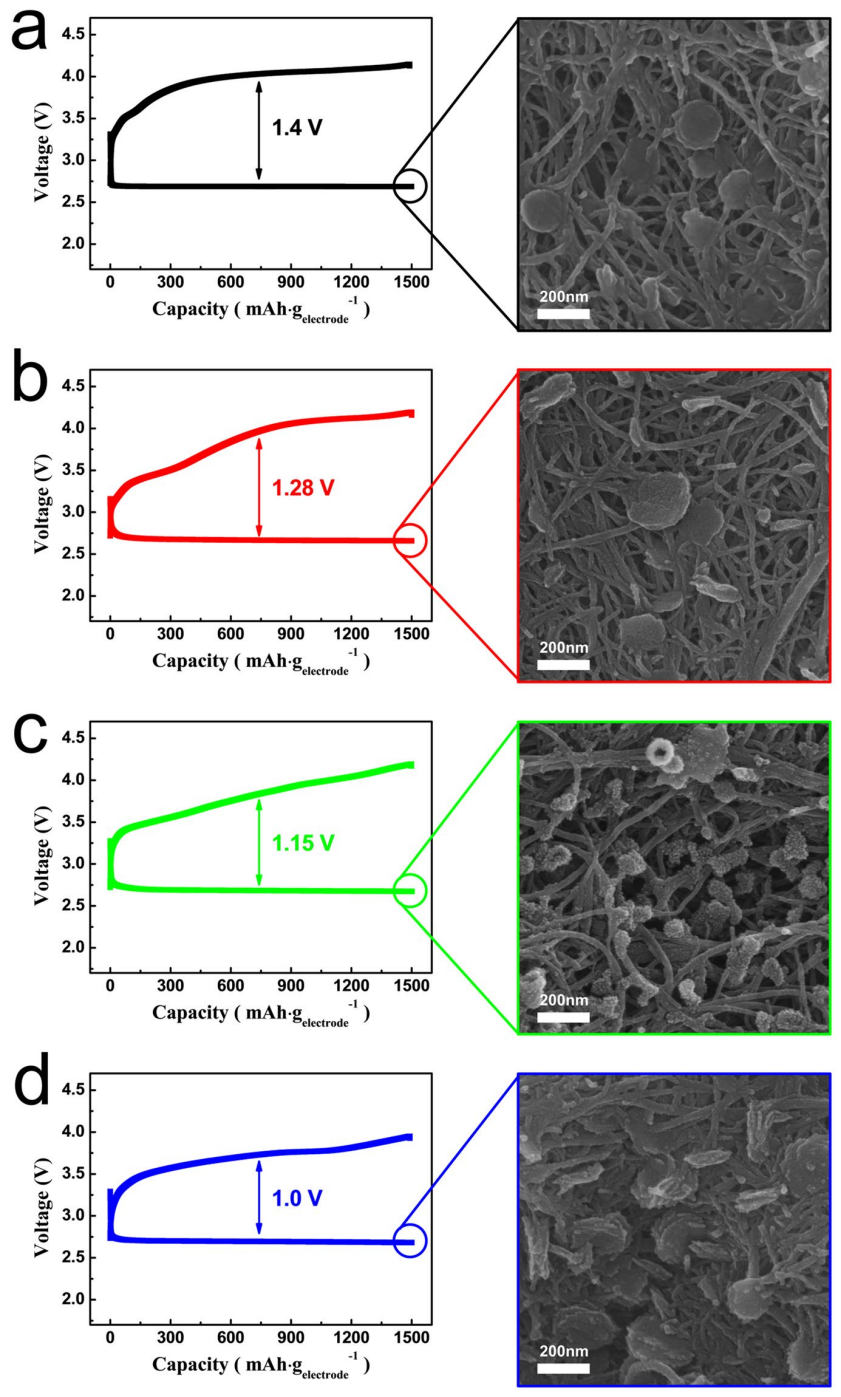
Initial discharge-charge profiles of the electrodes (left column) and SEM images of the surface morphologies of the discharged electrodes (right column). (**a**) The pristine electrode, (**b**) the PANI electrode, (**c**) the comp electrode, and (**d**) the PANI-comp electrode.

The effects of the PANI layer and the Co_3_O_4_ catalyst on the overpotential were also observed using the discharge-charge profiles of the cells measured with a capacity of 1500 mAh·g_electrode_^−1^. As shown in Fig. 3 (left column), the PANI and comp electrodes presented reduced overpotentials compared with that of the pristine electrode, which indicates that the reaction products can be easily dissociated at a somewhat lower potential range in the presence of the PANI layer and the Co_3_O_4_ catalyst. Although the catalytic activity of Co_3_O_4_ has been previously reported^45–47^, the PANI layer also appears to act as a catalyst by lowering the overpotential of the electrode. Considering that other conducting polymers such as PEDOT:PSS also exhibit redox activity in Li-air (Li-O_2_) cells^51^, the electronically conducting PANI layer plays a role in facilitating the dissociation of the reaction products. As shown in Fig. 3d, the overpotential of the PANI-comp electrode was considerably reduced compared to that of the pristine electrode due to the synergic effect of the PANI layer and the Co_3_O_4_ catalyst.

The right-hand column of Fig. 3 shows the morphologies of the different electrodes following initial discharge to the limited capacity of 1500 mAh·g_electrode_^−1^, which ultimately results in the formation of reaction products as a film or as particles on the electrode surface. Typically, the morphologies of such species are highly dependent on both the electrolyte solvent^59, 60^ and the current density^3^. However, in this case, the current density and solvent were maintained constant, and so any variations in the reaction product morphology were due to the electrode properties. As shown in the right-hand column of Fig. 3a, after discharge, the pristine electrode was coated with both film- and particle-type reaction products. Similar observations were made for the PANI electrode (Fig. 3b). However, the discharged comp electrode resulted in the formation of slightly smaller particles (Fig. 3c), which may be attributed to the effect of the Co_3_O_4_ catalyst. Furthermore, the reaction products on the surface of the discharged PANI-comp electrode appeared more abundant than those of the other electrodes. This can be explained by the PANI-comp electrode being in a more deeply discharged state due to its smaller full discharge capacity compared to the other electrodes, as shown in Figure S2. More specifically, the limited capacity of 1500 mAh·_gelectrode_^−1^ is approx. 35% of the full discharge capacity of the PANI-comp electrode, which is 13% of the full discharge capacity of the pristine electrode. In this case, the reaction products also appeared to be a mixture of film and particle types.

**Figure 4:**
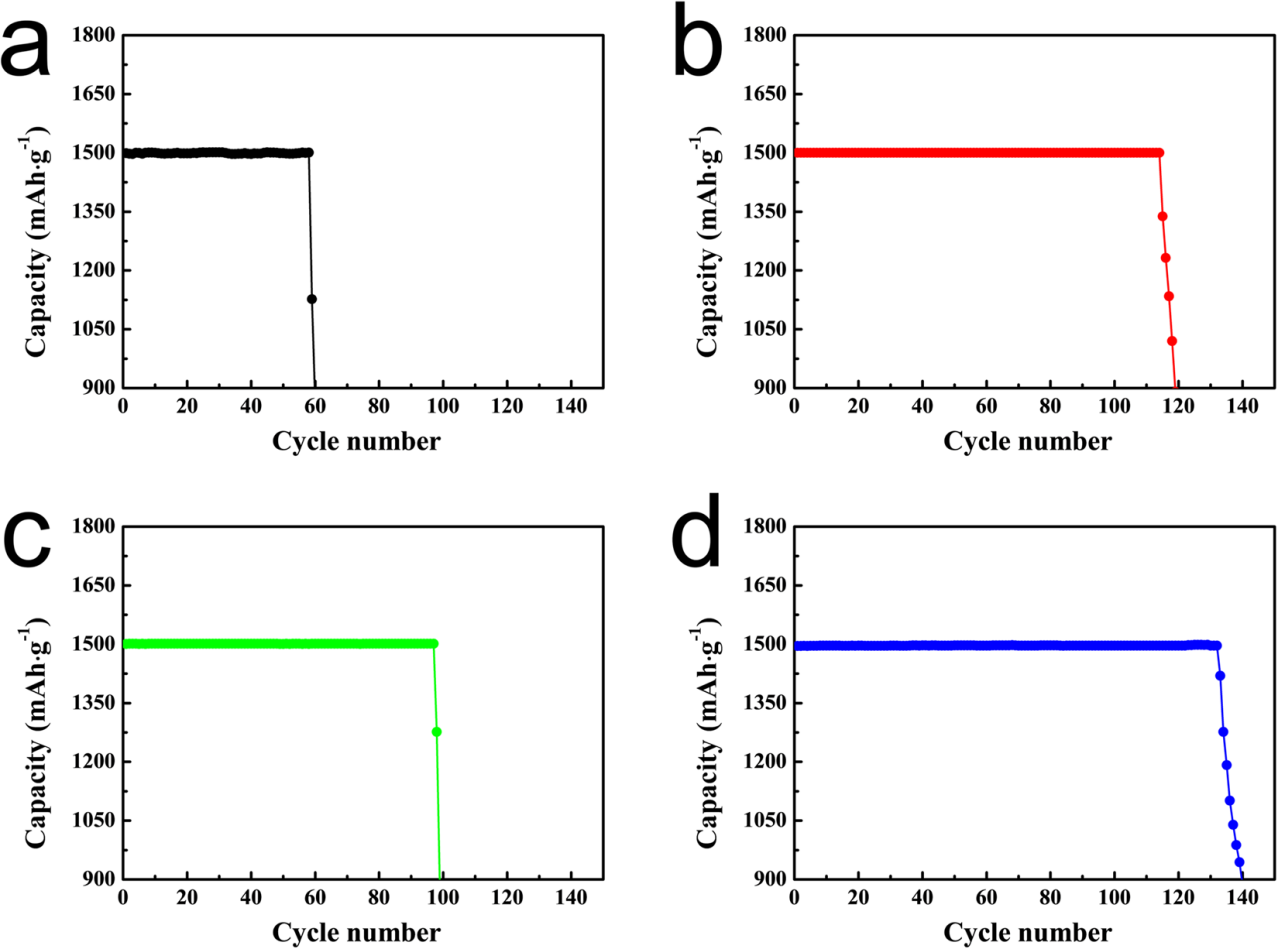
Cyclic performances of the electrodes at current densities of 500 mA·g_electrode_^−1^ (capacity = 1500 mAh·g_electrode_^−1^). (**a**) The pristine electrode, (**b**) the PANI electrode, (**c**) the comp electrode, and (**d**) the PANI-comp electrode.

The cycling performances of the cells employing the four different electrodes were then measured at current densities of 500 mA·g^−1^, and the cell capacities were set at 1500 mAh·g_electrode_^−1^ to prevent a large DOD (depth of discharge)^61^. As shown in Fig. 4, the cycle life of the pristine electrode was approximately 60 cycles under these measurement conditions, while the PANI electrode maintained a constant capacity over 114 cycles. This improved performance may be attributed to the PANI layer on the surface of CNTs suppressing any carbon-activated side reactions. In addition, the redox activity of PANI facilitates dissociation of the reaction products, which also contributes to the enhanced cyclic performance. The cycle life of the comp electrode was also superior to that of the pristine electrode, due to the high catalytic activity of the Co_3_O_4_ particles present on the CNT surface. Finally, the PANI-comp electrode exhibited a significantly enhanced cyclic performance compared with all other electrodes, maintaining a constant capacity beyond 130 cycles (134 cycles). Moreover, it should be considered that the electrodes were cycled with different DOD values, as their full discharge capacities differed (see Figure S2) despite a constant set-up capacity being employed (i.e., 1500 mAh·g_electrode_^−1^). Indeed, as previously mentioned, the DOD of the PANI-comp electrode (approx. 35% of full capacity) was significantly higher than that of the pristine electrode (approx. 13% of full capacity). Typically, a high DOD is unfavourable for achieving extended cycle lives. Nevertheless, the cycle life of the PANI-comp electrode was superior to those of the other electrodes, which exhibited lower DOD values. This enhanced cyclic performance can therefore be attributed to the synergic effect of the Co_3_O_4_ catalyst and the selectively-coated PANI layer. The changes in the discharge-charge profiles of the electrodes during the cycling process outlined in Fig. 4 can be observed in Figure S3. And the variation of average charging-potential values was presented in Figure S4.

Based on previous studies, we assumed that the cycle lives of the non-aqueous Li-air (Li-O_2_) cells would be affected by several factors, including the instability of the Li anode due to dendrite growth, consumption of the organic electrolyte through evaporation and side reactions, and the accumulation of reaction products on the air electrode promoting side reactions^4, 5, 9, 17^. Under the experimental conditions employed herein, both dendrite growth at the Li anode and electrolyte evaporation are uncontrollable. Thus, the cyclic performance of our electrodes was examined in the context of the side reactions taking place, as such processes result in the accumulation of unwanted products, including Li_2_CO_3_ and organic materials (e.g., CH_3_CO_2_Li and HCO_2_Li) on the surface of the air electrode, in addition to triggering decomposition of the organic electrolyte. Accordingly, suppression of the side reactions between the air electrode/organic electrolyte and the air electrode/Li_2_O_2_ interfaces would be expected to enhance the cycle performances of the Li-air (Li-O_2_) electrodes. Indeed, the preparation of selectively PANI-coated CNT/Co_3_O_4_ composites can be considered an effective approach to suppressing side reactions at the carbon surface at high potential ranges (i.e., >3.5 V)^28–31^. More specifically, the Co_3_O_4_ catalyst can facilitate dissociation of the reaction products responsible for reducing the overpotential and lowering the potential range during the charging process. In addition, the PANI layer prevents direct contact between the carbon (CNT) electrode and the electrolyte and/or Li_2_O_2_ without affecting the Co_3_O_4_ catalytic activity, as it is selectively coated on the CNT surface.

**Figure 5:**
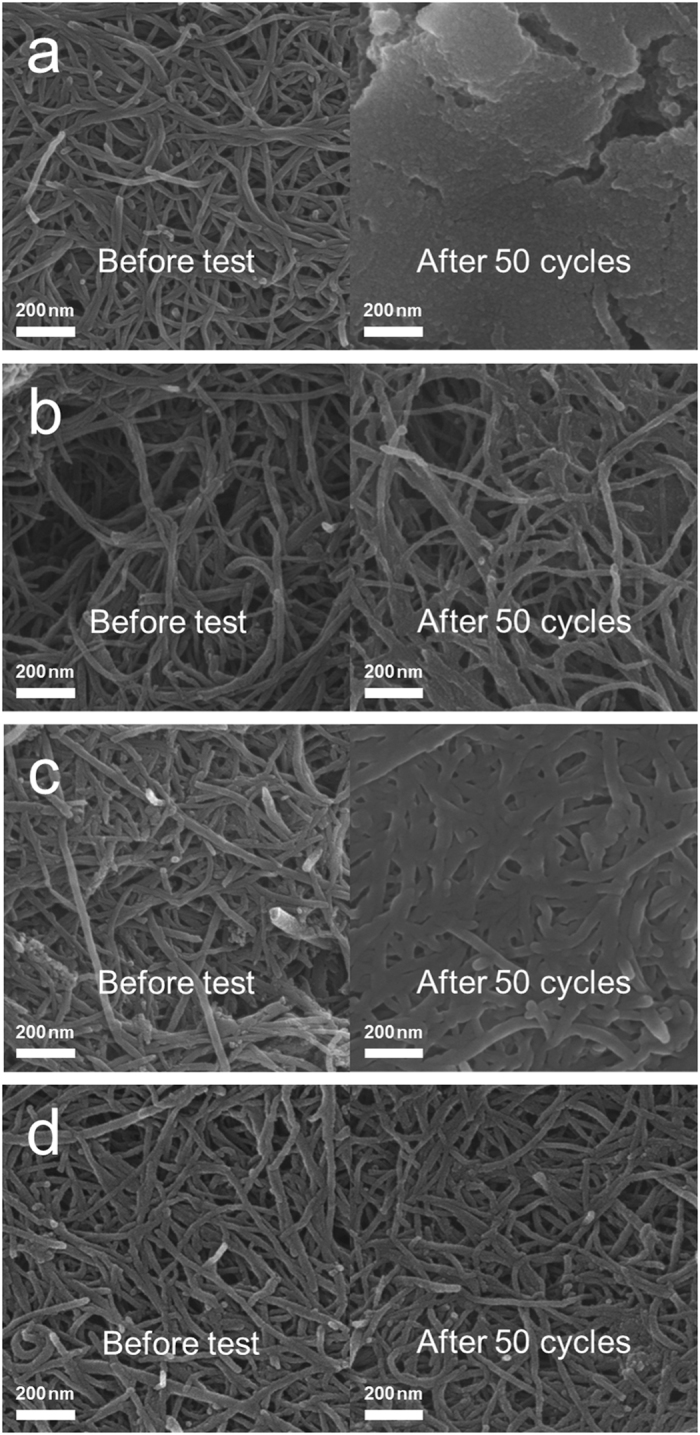
SEM images of the electrodes after 50 cycles (charged state). The cells were cycled at a limited capacity of 1500 mAh·g_electrode_^−1^. (**a**) The pristine electrode, (**b**) the PANI electrode, (**c**) the comp electrode, and (**d**) the PANI-comp electrode.

To determine the effects of the PANI layer and the Co_3_O_4_ catalyst in more detail, the four air electrodes were analysed both before and after cycling using SEM and FTIR spectroscopy. Figure 5 shows the SEM images of the pristine, PANI, comp, and PANI-comp electrodes before the test and after 50 cycles (charged state). As shown in the left-hand column of the figure, all electrodes exhibited a fibrous CNT structure prior to testing. However, after cycling, despite its charged state, the pristine electrode was covered with residual reaction products (Fig. 5a). In contrast, although a small quantity of reaction products was observed on the surface of the PANI electrode after cycling, the fibrous texture was maintained (Fig. 5b), indicating that the PANI layer effectively suppresses the accumulation of reaction products. As shown in Fig. 5c, the cycled comp electrode also contained only smaller residual quantities of reaction products compared to the pristine electrode. This is likely due to the high catalytic activity of the Co_3_O_4_ particles, which assist dissociation of the reaction products. However, the amount of residual reaction products was somewhat larger than that observed for the PANI electrode. Notably, the cycled PANI-comp electrode clearly displayed a fibrous texture and porous structure, which supply sufficient vacant spaces to allow the Li ions and oxygen to reach the catalytic sites on the electrode surface. This result clearly indicates that the use of a selectively PANI-coated CNT/Co_3_O_4_ composite is effective for reducing the quantities of residual reaction products present on the surface during and following cycling.

**Figure 6:**
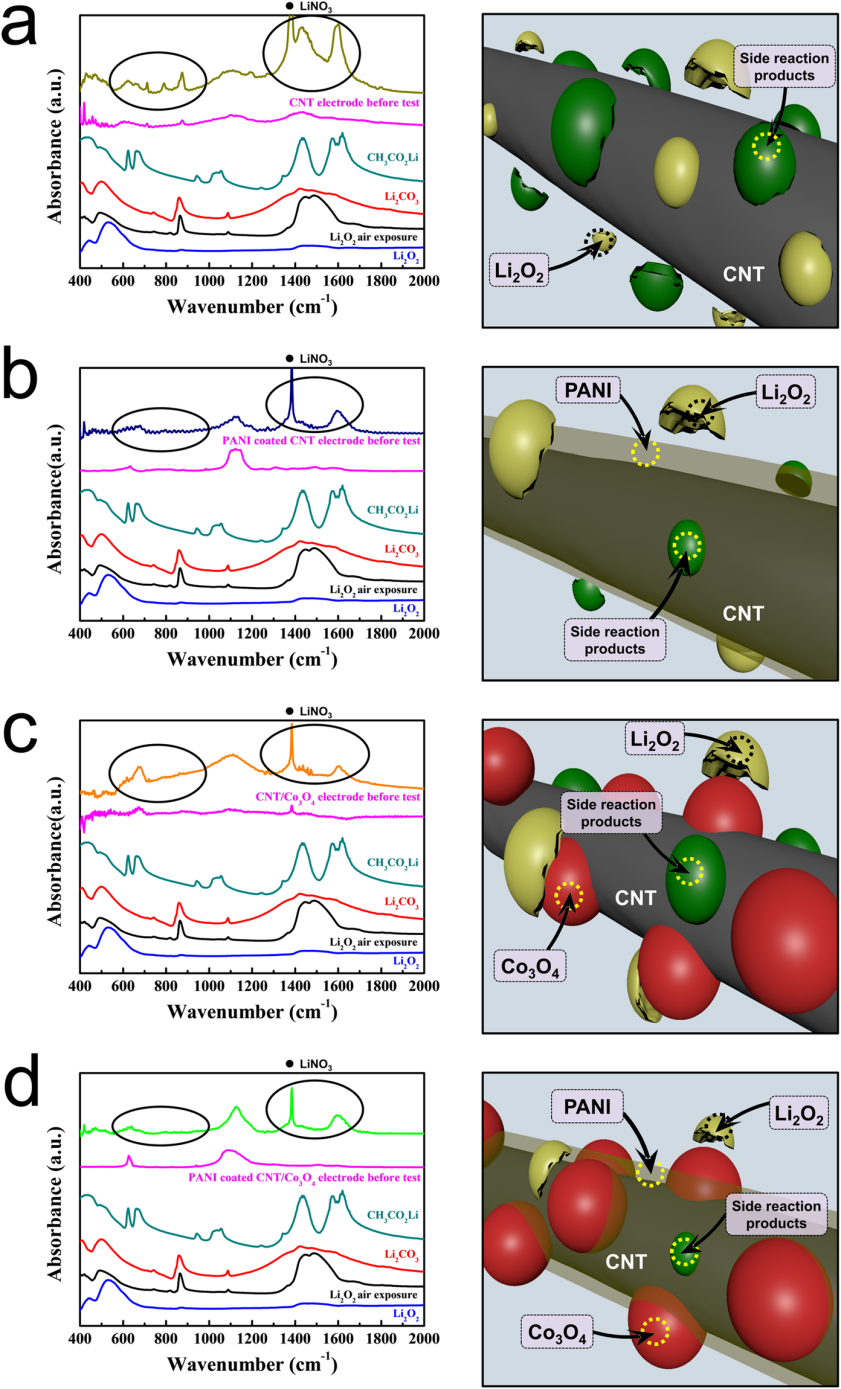
FTIR spectra of the electrodes (left column) after 50 cycles (charged state) and illustrations (right column) of the reactions taking place on the electrode surfaces. (**a**) The pristine electrode, (**b**) the PANI electrode, (**c**) the comp electrode, and (**d**) the PANI-comp electrode.

The FTIR spectra of the four electrodes recorded before testing and after 50 cycles (charged state) are given in the left-hand column of Fig. 6. As shown in Fig. 6a, the cycled pristine electrode exhibited large peaks at 1,350–1,500 and 1,500–1,700 cm^−1^ in addition to several smaller peaks at 600–900 cm^−1^. These peaks were attributed to unwanted reaction products, such as Li_2_CO_3_ and CH_3_CO_2_Li, which were derived form side reactions between the electrode and Li_2_O_2_ and/or the electrolyte.

**Figure 7:**
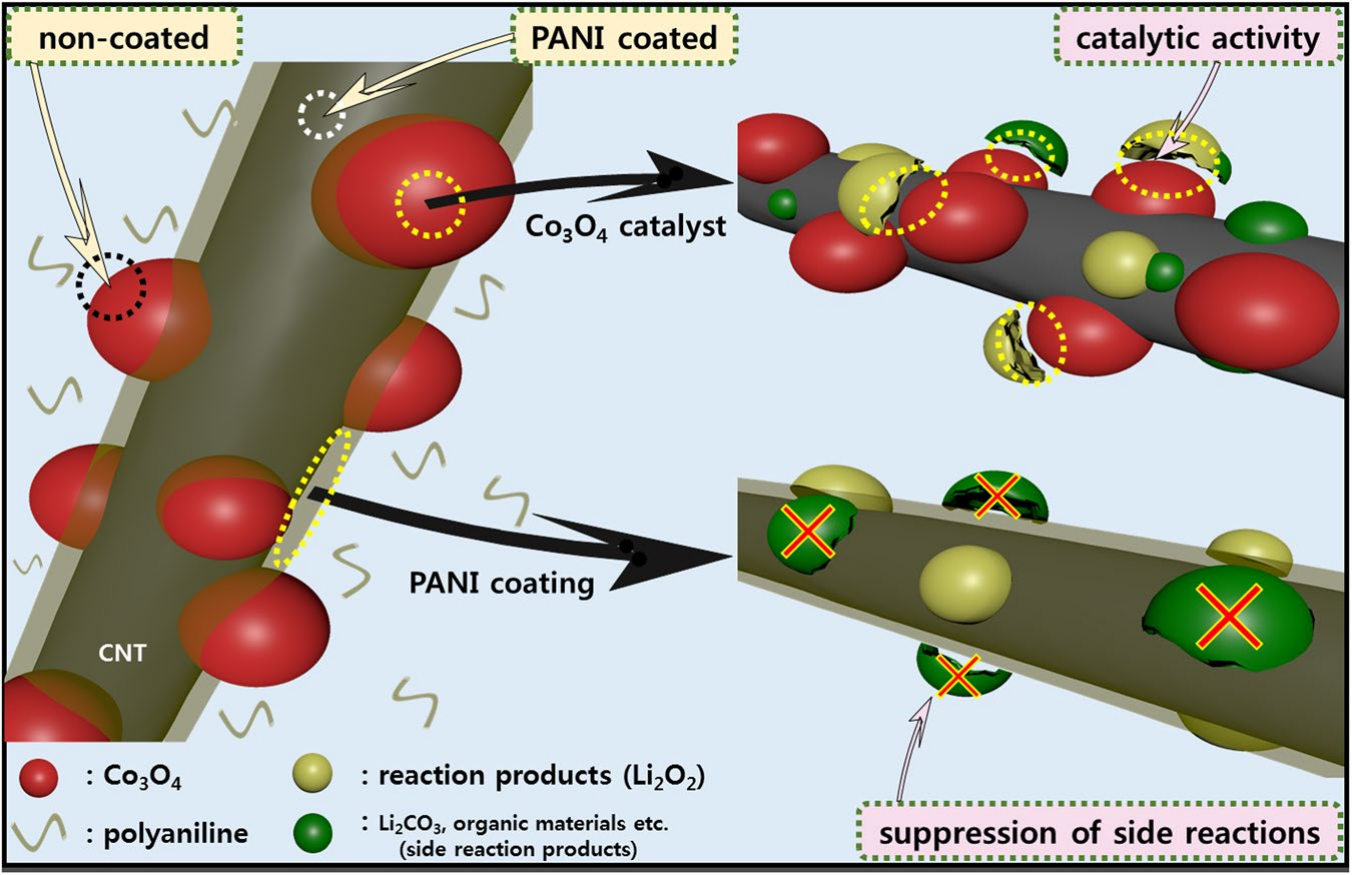
Schematic illustration demonstrating the synergic effect of the PANI layer and the Co_3_O_4_ catalyst particles in the selectively PANI-coated CNT/Co_3_O_4_ nanocomposites.

This result indicates that the majority of residual reaction products present on the pristine electrode after cycling originated from this side reaction. In contrast, the peak intensities of these undesirable reaction products significantly decreased for the cycled PANI electrode (Fig. 6b), which indicates that the PANI layer can suppress this side reaction. The right-hand column of Fig. 6 shows illustrations of the reactions taking place on the electrode surface. As shown, during cycling, the pristine electrode becomes coated with unwanted reaction products derived from the side reactions, which clogs the electrode and limits its cycle performance (Fig. 6a). In contrast, the PANI electrode can suppress such side reactions through protection of the carbon surface (Fig. 6b). In the spectrum of the cycled comp electrode, the signal intensities corresponding to the unwanted reaction products were lower than those observed for the pristine electrode, as shown in Fig. 6c. However, the quantity of unwanted reaction products seemed somewhat larger compared to the PANI electrode. This suggests that the PANI layer is more efficient in suppressing the side reactions than the Co_3_O_4_ catalyst, despite its high catalytic activity, as the addition of catalyst particles results in the majority of the carbon surface remaining in contact with Li_2_O_2_ and with the electrolyte. Thus, as shown in the illustration of Fig. 6c, a considerable quantity of reaction products accumulated on the comp electrode surface during cycling. Finally, the FTIR spectrum of the PANI-comp electrode exhibited only small peaks corresponding to the unwanted side products (Fig. 6d), likely due to a combination of the PANI layer and the Co_3_O_4_ catalyst. This effect therefore contributes to the good cyclic performance of the composite, as illustrated in Fig. 4d. Thus, from the SEM and FTIR results, we could conclude that the superior cyclic performance and low over-potential of this electrode were attributed to the synergic effect of the PANI layer (suppression of side reactions) and the Co_3_O_4_ particles, thereby leading to a high catalytic activity. These observations are summarized in Fig. 7.

## Conclusion

In summary, we successfully prepared carbon nanotube (CNT)/Co_3_O_4_ nanocomposites selectively coated with polyaniline (PANI) via an electropolymerization method for application as an effective electrode material for Li-air (Li-O_2_) batteries. Although the carbon surface of the CNTs was homogeneously coated by a PANI layer, the Co_3_O_4_ particles remained uncoated, thus allowing them to retain their catalytic activity. The presence of this PANI layer on the CNT surface effectively reduced the overpotential of the electrode, which indicates that it exhibits a degree of catalytic activity. In addition, the cell employing the selectively-coated CNT/Co_3_O_4_ nanocomposite electrode exhibited a superior electrochemical performance. More specifically, the Co_3_O_4_ particles present on the CNT surface facilitates the dissociation of reaction products such as Li_2_O_2_, whilst also reducing the overpotential. In addition, the presence of this PANI layer on the carbon surface limits direct contact with Li_2_O_2_ and with the electrolyte, thus suppressing side reactions involving these species. As a result, the cell containing the selectively-coated CNT/Co_3_O_4_ nanocomposites exhibited enhanced cycle performances and lower overpotentials than those based on pristine CNTs, PANI-coated CNTs, and CNT/Co_3_O_4_ nanocomposites due to the synergic effect of the Co_3_O_4_ catalyst particles and the PANI layer. We therefore expect that these results will contribute to the development of novel electrodes for Li-air (Li-O_2_) batteries exhibiting enhanced cycle performances.

## Methods

### Synthesis of the selectively PANI-coated CNT/Co_3_O_4_ composite

Figure S5 outlines the procedure employed for synthesis of the selectively PANI-coated CNT/Co_3_O_4_ nanocomposites. The CNT/Co_3_O_4_ composite was initially prepared followed our previously reported method^46^. At first, polydopamine coated CNTs was prepared using dopamine solution containing a tris-buffer solution (10 mM, pH 8.5) and methanol as co-solvents (CH_3_OH:buffer = 1:1 v/v). Then, the polydopamine-coated CNTs were reacted with Co(NO_3_)_2_∙6H_2_O (cobalt nitrate hexahydrate) aqueous solution. Finally, that was annealed under air at 450 °C for 4 h to obtain final nanocomposites. The PANI layer was subsequently applied using an electropolymerization method on the surface of CNT/Co_3_O_4_ nanocomposites. More specifically, a platinum wire (5 cm) and a saturated calomel electrode (SCE) were employed as the counter and reference electrodes, respectively, at room temperature. The CNT/Co_3_O_4_ electrode (5 cm × 3 cm) was directly employed as the working electrode for the deposition of PANI. This electrode was prepared by mixing the CNT/Co_3_O_4_ electrode (90 wt.%) with a polyvinylidene fluoride (PVDF) binder (10 wt.%). The loading weight was adjusted to 0.3 mg ± 0.03 mg. The aniline solution was prepared by mixing a 1 M aniline solution (monomer, Sigma-Aldrich) with a 1 M NaClO_4_ solution prepared in deionized water with stirring over 2 h. The CNT/Co_3_O_4_ electrode was then immersed in the aniline solution for electropolymerization. Cyclic voltammetry was carried out between −0.3 and +1.2 V vs. SCE by scanning the potential at a scan rate of 50 mVs^−1^ for 3 cycles. The coated sample was then dried in an oven at 60 °C for 24 h. For comparison, PANI-coated CNTs were also prepared using the above method.

### Electrochemical tests

The electrochemical performances of the electrodes were measured using a Swagelok cell consisting of an air electrode, a metallic Li anode, glass fibre filter paper (as a separator, 0.7 μm), and an electrolyte containing 0.5 M LiTFSI and 0.5 M LiNO_3_ dissolved in tetraethylene glycol dimethyl ether (TEGDME). The cells were assembled in an Ar-filled glove box and were subjected to galvanostatic cycling using a WonATech battery cycler (WBCs 3000). In all electrochemical experiments, the current density and voltage range were measured at 500 mA·g_electrode_^−1^ and 2.0–4.35 V, respectively. The capacity was determined based on the total electrode mass (active material + binder). For the cycling test, the capacity was limited to 1500 mAh·g_electrode_^−1^ to prevent a large depth-of-discharge (DOD). All experiments were conducted under an O_2_ atmosphere at ambient pressure.

### Characterization of the electrode

Transmission electron microscopy (TEM, JEM-2100F (HR), JEOL/CEOS) was employed to examine the morphologies of the pristine CNTs, the PANI-coated CNT, the CNT/Co_3_O_4_ composite, and the selectively-coated CNT/Co_3_O_4_ composite. In addition, scanning electron microscopy (SEM, Nova NanoSEM 450, FEI Co.) was employed to examine the surface morphologies of the air-electrodes in greater detail. Furthermore, Fourier transform infrared spectroscopy (FT-IR, Nicolet 5700, Thermo Electron Corp.) was performed to confirm formation of the PANI layer, in addition to determining reaction product accumulation during cycling.

### Data availability

All data generated or analyzed during this study are included in this published article (and its Supplementary Information files).

## Electronic supplementary material

Supporting Information
