# Supplementary material for: Carbon nanotube/Co3O4 nanocomposites selectively coated by polyaniline for high performance air electrodes
Source: Sci Rep. 2017 Aug 17;7:8610. doi: 10.1038/s41598-017-09219-9 (PMC5561172; doi:10.1038/s41598-017-09219-9)
Supplement: Supplementary file 1 — Supporting Information [file 41598_2017_9219_MOESM1_ESM.pdf]

# Supporting Information

## Carbon nanotube/$Co_3O_4$ nanocomposites selectively coated by polyaniline for high performance air electrodes

**Jin Young Kim and Yong Joon Park***

*Department of Advanced Materials Engineering, Kyonggi University, 154-42, Gwanggyosan-Ro, Yeongtong-Gu, Suwon-Si, Gyeonggi-Do, 443-760, Republic of Korea*
*Email: yjpark2006@kyonggi.ac.kr*

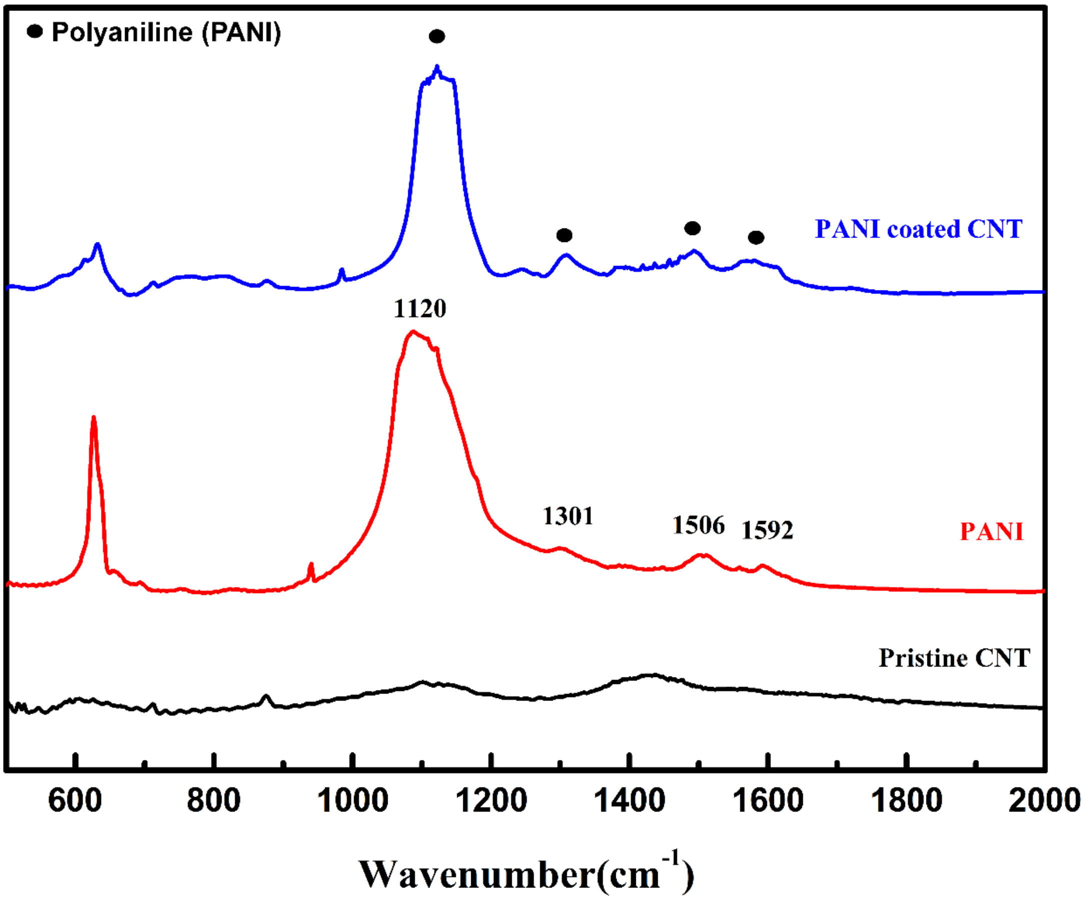


**Figure S1.** FTIR spectra of the pristine CNTs, PANI, and the PANI-coated CNT/$Co_3O_4$ nanocomposite.

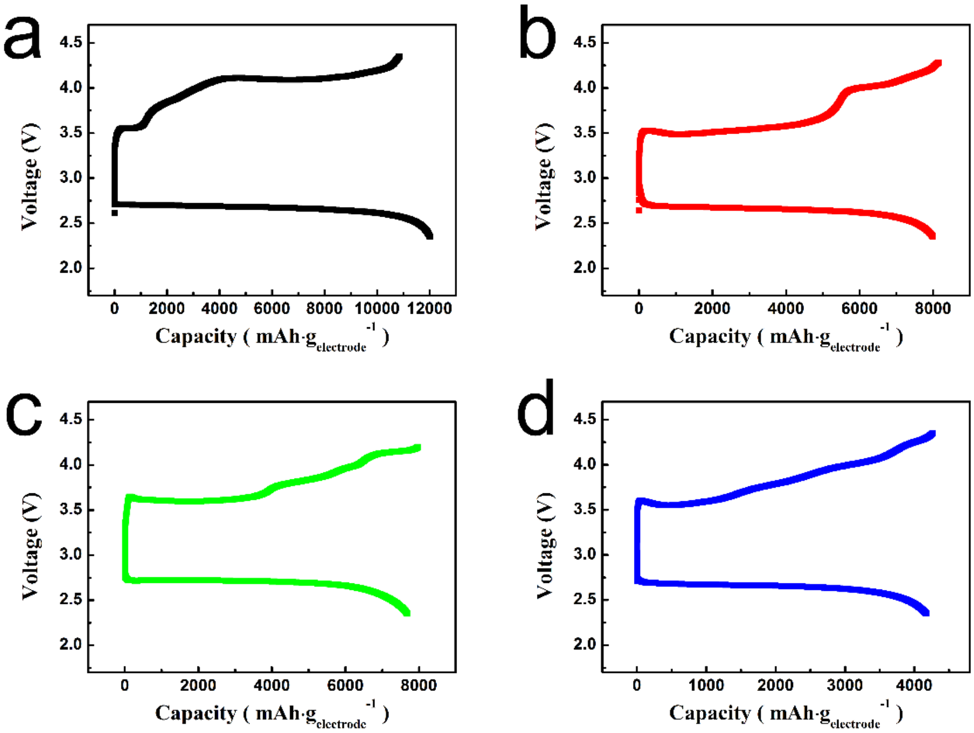


**Figure S2**. Initial full discharge-charge profiles of the different electrodes. (a) The pristine electrode, (b) the PANI electrode, (c) the comp electrode, and (d) the PANI-comp electrode.

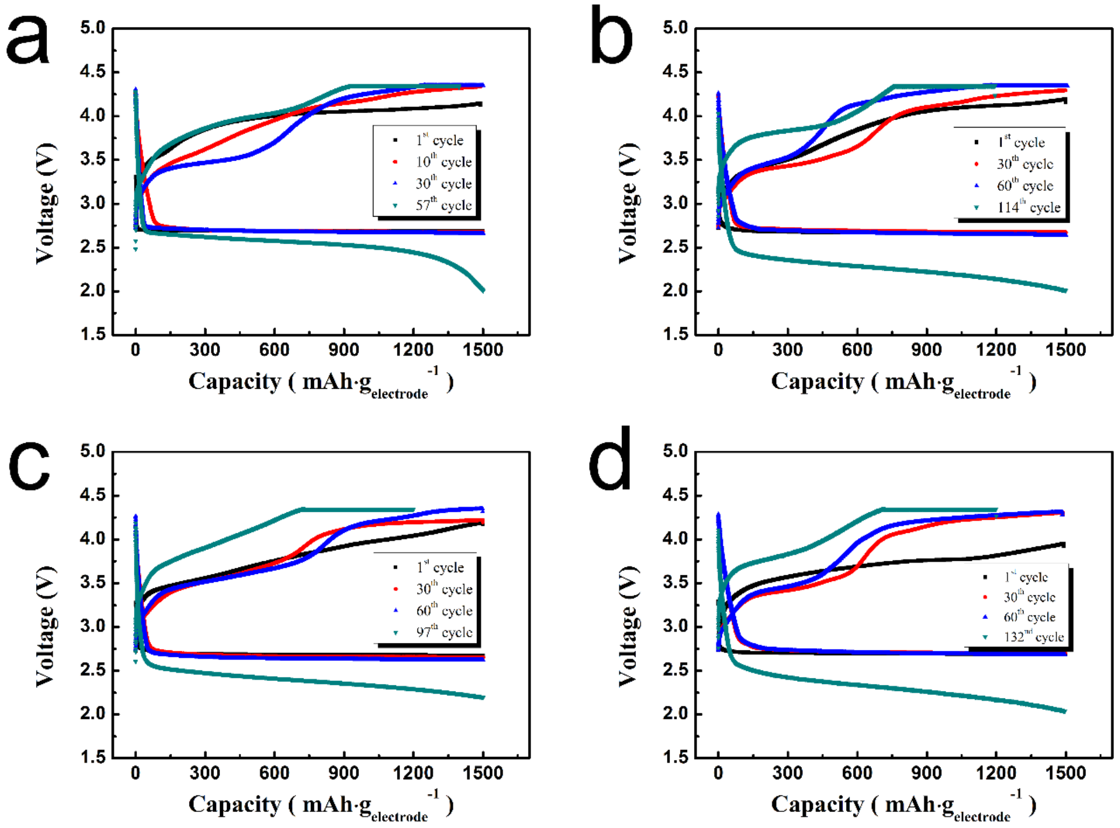


**Figure S3.** Variations in the discharge-charge profiles of the differences electrodes during cycling at a limited capacity of 1500 mAh·g$^{-1}$. (a) The pristine electrode, (b) the PANI electrode, (c) the comp electrode, and (d) the PANI-comp electrode.

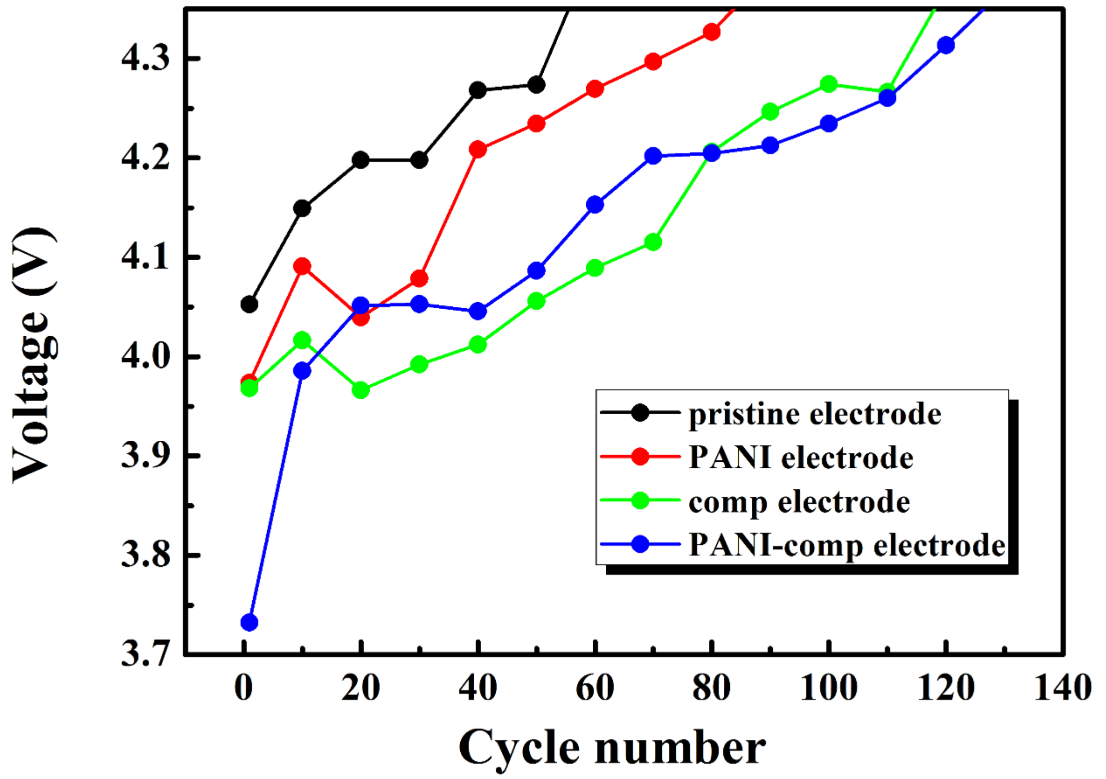


**Figure S4.** Average charging-potential of the different electrodes during cycling at a limited capacity of 1500 mAh·g$^{-1}$. (a) The pristine electrode, (b) the PANI electrode, (c) the comp electrode, and (d) the PANI-comp electrode.

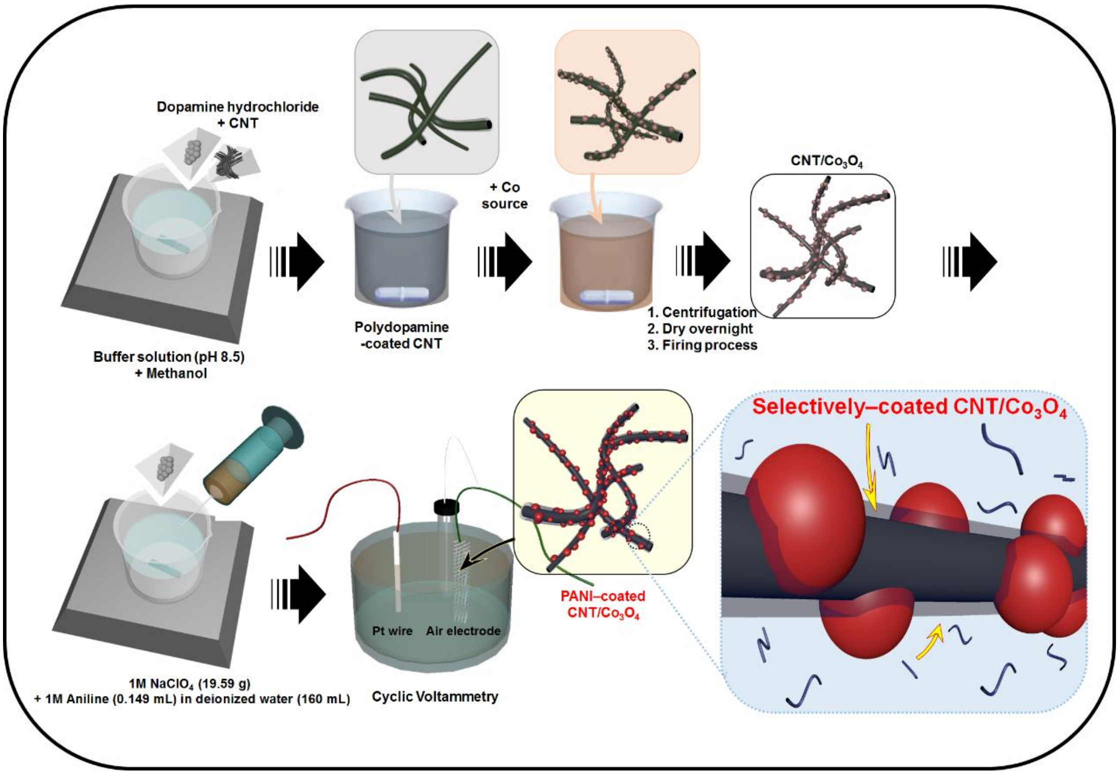


**Figure S5**. Schematic illustration showing preparation of the selectively PANI-coated CNT/$Co_3O_4$ nanocomposites.
